# Genomic Surveillance Uncovers the Silent Spread of Avian Influenza Virus (H5N1 2.3.4.4b) Among Wild Birds and Mammals Along Brazil’s Southern Coast

**DOI:** 10.3390/v18070738

**Published:** 2026-07-03

**Authors:** Yasmin Luisa Neves Lemes Garcia, Fábio Henrique de Lima, Dayla Bott Geraldini, Ana Júlia Chaves Gomes, Isabella do Vale Francisco Bortolato, Eliana Leonor Hurtado Celis, Guilherme Guerra Neto, Natasha Fujii Ando, Camila Sanches Rodrigues, Richard Alegria Cesario, Cecília Artico Banho, Helena Lage Ferreira, João Pessoa Araújo Junior, Maurício Lacerda Nogueira, Fernando Rosado Spilki, Edison Luiz Durigon, Danielle Bruna Leal Oliveira, Camila Domit, Vivaldo Gomes da Costa, Marília Freitas Calmon, Paula Rahal

**Affiliations:** 1 Laboratório de Estudos Genômicos, Departamento de Biologia, Universidade Estadual Paulista (UNESP), São José do Rio Preto 15054-000, SP, Brazil; yasminlnlgarcia@gmail.com (Y.L.N.L.G.); dayla.geraldini@unesp.br (D.B.G.); aj.gomes@unesp.br (A.J.C.G.); isabella.bortolato@unesp.br (I.d.V.F.B.); 2 Laboratório de Ecologia e Conservação, Universidade Federal do Paraná (UFPR), Pontal do Paraná 83255-976, PR, Brazil; fabiopmpparana@gmail.com (F.H.d.L.); cadomit@gmail.com (C.D.); 3 Laboratório de Virologia e Diagnóstico Molecular, Instituto de Biotecnologia, Universidade Estadual Paulista (UNESP), Botucatu 01049-010, SP, Brazil; eliana.hurtado@unesp.br (E.L.H.C.); joao.pessoa@unesp.br (J.P.A.J.); 4 Jardim Zoobotânico de São José do Rio Preto, São José do Rio Preto 15053-600, SP, Brazil; guigulo@gmail.com (G.G.N.); natasha.fujii@gmail.com (N.F.A.); camillasanro@gmail.com (C.S.R.); rialegria.medvet@gmail.com (R.A.C.); 5 Laboratório de Pesquisa em Virologia, Faculdade de Medicina de São José do Rio Preto (FAMERP), São José do Rio Preto 15090-000, SP, Brazil; ceci.abanho@gmail.com (C.A.B.); mauricio.nogueira@edu.famerp.br (M.L.N.); 6 Laboratório de Medicina Veterinária Preventiva Aplicada, Departamento de Medicina Veterinária, Faculdade de Zootecnia e Engenharia de Alimentos, Universidade de São Paulo (USP), São Paulo 05508-220, SP, Brazil; hlage@usp.br; 7 Department of Pathology, University of Texas Medical Branch, Galveston, TX 77555-0609, USA; 8 Laboratório de Microbiologia Molecular, Universidade Feevale, Novo Hamburgo 93525-075, RS, Brazil; fernandors@feevale.br; 9 Laboratório de Virologia Clínica e Molecular, Departamento de Microbiologia, Universidade de São Paulo (USP), São Paulo 05508-220, SP, Brazil; eldurigo@usp.br (E.L.D.); danibruna@usp.br (D.B.L.O.); 10 Hospital Israelita Albert Einstein, São Paulo 05652-900, SP, Brazil

**Keywords:** H5N1, avian Influenza, mammals, birds, genomic surveillance

## Abstract

Avian influenza viruses (AIVs) are widely distributed and have a wide range of hosts. Recently, the number of cases of infection associated with the circulation of highly pathogenic avian influenza H5N1 2.3.4.4b has raised concerns about its high transmission capacity in birds and mammals. This study analyzed swabs from bird and mammal species from the coast of Paraná and the northwest region of São Paulo, Brazil, for the presence of AIV in animals that did not present clinical or histopathological lesions of infection that indicated the need for molecular characterization during monitoring. Of the 661 animals analyzed, three tested positive, two of which were birds (*Sula leucogaster* and *Thalasseus acuflavidus*) while one was a mammal (*Otaria flavescens*) (0.45%, CI 95%: 0.16–1.33). A complete genome sequence of H5N1 AIV was obtained from a brown booby (*Sula leucogaster*) from the Paraná coast (GISAID accession number: EPI_ISL_1897537). Our study reinforces the importance of continuous genomic surveillance, especially in AIV hosts that do not show signs of infection, to enhance the One-Health assessment approach.

## 1. Introduction

Avian influenza viruses (AIVs) are widely distributed in nature. They can be isolated from various domestic and wild animals, including birds, sea lions, humans, pigs, dogs, and cats [1]. Over the last few years, there has been an evident increase in the number of cases associated with the circulation of the highly pathogenic AIV (HPAIV) H5N1 clade 2.3.4.4b, along with documented human infections and outbreaks in avian species, which reinforce concerns regarding zoonotic transmission [2,3,4,5]. Wild birds infected with the virus may have led to spillover events affecting mammals that cohabitate in the same habitat areas [6]. Evidence suggests that there has been a change in the epidemiological behavior of the virus, meaning that it is now able to infect a large number of birds and other unusual hosts [7].

AIVs are members of the *Orthomyxoviridae* family, measuring between 80 and 120 nm in diameter. They consist of a host-derived lipid envelope containing the glycoproteins hemagglutinin (HA), Neuraminidase (NA), as well as the membrane protein (M2) and the Matrix protein (M1) beneath the envelope, and the ribonucleoproteins, which are genomic structures composed of the eight-segment negative-sense viral RNA genome associated with nucleoprotein (NP) [8]. The eight segments of viral RNA encode 10 major gene products, the polymerase proteins PB1, PB2, and PA, the glycoproteins (HA and NA), the NP, M1 and M2 proteins and the non-structural proteins (NS1 and NS2) [8,9]. The HA and NA glycoproteins are located on the viral surface and exhibit a high mutation rate [10].

Recently, phylogenetic analyses of the HA gene have indicated that HPAIV H5N1 (2020–2023) strains in South America originated from multiple introductions from North America, particularly via the Pacific Flyway [11]. This virus can be traced back to the original strain A/goose/Guangdong/1/96, which was first isolated in Hong Kong, China, in 1997 [12,13]. In Brazil, the first case of HPAIV H5N1 was detected in May 2023, in two specimens of the migratory Cabot’s Tern (*Thalasseus acuflavidus*) in the city of Marataízes in the state of Espírito Santo [5]. Alongside the mortality of wild and domestic birds, several sea lions exhibiting neurological and respiratory signs, and ultimately succumbing, were observed on beaches [14,15].

Recent data shows a significant increase in HPAIV H5N1 2.3.4.4b cases spreading from birds to mammals. Additionally, studies have pointed out that mammal-to-mammal transmission contributes to outbreaks among dairy cows in North America, fur farms in Finland, and human infections in Ecuador and Chile [16,17,18,19,20,21,22]. The virus has been circulating among wild birds for years, causing substantial economic losses in the poultry industry and zoonotic infections [21]. This fact has led to increased concern regarding the pandemic potential of the virus and highlights the need for a more comprehensive understanding of its characteristics and public health impact. This case reinforces the relevance of the One Health approach and the urgent need to integrate environmental and health sciences. Therefore, this study, initiated before the confirmation of the first HPAIV H5N1 2.3.4.4b cases in Brazil, aimed to implement a surveillance system focused on the early detection of the virus in wild animals that were not classified as suspected cases during wildlife monitoring because they did not exhibit the clinical signs or histopathological findings typically considered indicative of AIV infection at the time of evaluation. The proposal was motivated by the increasing number of HPAIV detections across the Americas in late 2021 [23]. Active surveillance was considered essential to identify potential undetected viral circulation, reinforcing the importance of preventive actions involving monitoring and epidemiological investigation.

## 2. Materials and Methods

### 2.1. Ethics Statement

Samples from the northwestern region of São Paulo were obtained from the São José do Rio Preto Zoobotanical Garden, while samples from the Paraná coast were provided by the Laboratório de Ecologia e Conservação of the Universidade Federal do Paraná (UFPR). The animal procedures were approved by the Animal Care Committee of the Universidade de São Paulo, Pirassununga, Brazil (approval ID: 001686). The samples collected by the UFPR were obtained as part of the Santos Basin Beach Monitoring Project (PMP-BS, “Projeto de Monitoramento de Praias da Bacia de Santos”). This project was required by the Brazilian federal environmental agency (IBAMA) for the environmental licensing of PETROBRAS. IBAMA Authorization for the Capture, Collection, and Transport of Biological Material (ABIO No. 640/2015, valid until 12 August 2027).

### 2.2. Biological Samples

Analysis was conducted at the Laboratório de Estudos Genômicos at Universidade Estadual Paulista “Júlio de Mesquita Filho”, in São José do Rio Preto, São Paulo, Brazil. The samples came from two Brazilian states, São Paulo and Paraná, from July 2022 to May 2025. The São José do Rio Preto Zoobotanical Garden collected oropharyngeal and cloacal swab samples from live avian species rescued from 37 cities in the northwest region of São Paulo state (SP) for clinical and veterinary treatment. The animals were rescued by environmental agencies, found in irregular places, victims of trafficking, run over, burned, or orphaned chicks. After being treated, they were released back into their habitats or sent to shelters, if possible.

Additionally, samples of birds and mammals from the coast of Paraná (PR) were sent by Laboratório de Ecologia e Conservação of the UFPR. These animals are monitored as part of the Santos Basin Beach Monitoring Project (PMP-BS). The swab samples varied greatly, and included samples from oral, anal, nasal, pulmonary, cerebral, and other organs, depending on the type of animal sampled and its condition, ranging from live animals (considered a 1 on the scale) to those at different stages of decomposition (scale of 2–4) [24].

### 2.3. Histopathology

All animals were tested and their tissue samples were stained using the hematoxylin–eosin method. The tissues were analyzed, depending on the decomposition stage, including the adrenal glands, spleen, cerebellum/brain, heart and large vessels, stomach, liver, gonads, small intestine, large intestine, skeletal muscle, pancreas, skin, lungs, kidneys, and thyroid and parathyroid glands.

### 2.4. RNA Extraction

RNA extraction was conducted using TRIzol^®^ Reagent method (Invitrogen^®^, Carlsbad, CA, USA), according to the manufacturer’s instructions, with modifications. For this purpose, 250 μL swab samples were added to 750 μL of TRIzol^®^, and after 5 min of incubation, 200 μL of chloroform (Merck Millipore^®^, Burlington, MA, USA) was added to the mixture, followed by 15 min of centrifugation at 4°C (14,000 rpm). In another tube, the aqueous phase was transferred, 500 μL of absolute isopropanol (Merck Millipore^®^, USA) was added, and the mixture was incubated for 10 min and then centrifuged for 20 min (14,000 rpm). Then, once the pellet was formed, the liquid content was discarded and the tube containing the total RNA pellet was washed with 75% DEPEC ethanol (Merck Millipore^®^, USA) and centrifuged one last time at a lower speed, 10,000 rpm for 10 min. After the liquid was discarded, the pellet was dried under a vacuum and resuspended in 30 μL of DEPC-treated water (Sigma-Aldrich^®^, St. Louis, MO, USA). In the final step, the extracted material was quantified using a NanoDrop 2000 spectrophotometer (Thermo Fisher Scientific^®^, Waltham, MA, USA). After that, the samples were stored at −80 °C; they were kept frozen until subsequent analyses.

### 2.5. RT-qPCR for AIV Detection

Real-Time qPCR reactions were performed on the QuantStudio 12K Flex system (Applied Biosystems, Foster City, CA, USA) using the AgPath-ID™ One-Step Kit (Thermo Fisher Scientific, Waltham, MA, USA) with specific primers and probes targeting the Matrix gene, as described by Spackman et al. [25]. The cycling conditions were as follows: denaturation at 45 °C for 10 min and 95°C for 5 min, followed by 45 cycles of annealing and extension at 94 °C for 10 s, 57 °C for 30 s, and 72 °C for 10 s.

All samples were analyzed by RT-qPCR using an endogenous control to verify the sample quality. Therefore, primers and probe targeting the beta-actin gene were used [26]. The cycling conditions were as follows: denaturation at 45 °C for 10 min and 95 °C for 10 min, followed by 45 cycles of 95 °C for 15 s and 60 °C for 1 min for annealing and extension. All analyses were performed in linear and multi-component mode, adjusting the baseline to the cycle threshold (Ct) of positive and negative controls to eliminate possible reagent interference. Samples with Ct values < 40 were considered positive.

### 2.6. Virus Isolation in Embryonic Eggs

At the BSL 3 Laboratory at the Universidade de São Paulo, positive lung and brain samples from the Patagonian sea lion were inoculated into chicken embryo eggs for replication. Initially, the samples were centrifuged at 3000× *g* for 30 min, after which the collected supernatant was passed through a membrane filter (0.22 μm). Then, 0.2 mL was inoculated into the allantoic cavity of a 10-day-old specific pathogen-free embryonated chicken egg and incubated for 72–96 h at 37 °C. The allantoic fluid from the samples was passed twice into the embryonated egg and was then tested by RT-qPCR for viral presence [27], as previously described. However, virus isolation was unsuccessful, possibly due to the low viral load and/or the advanced state of carcass decomposition.

### 2.7. Next-Generation Sequencing (NGS)

The positive swab sample was processed using the Total RNA Purification Kit (Norgen Biotek Corporation, Thorold, ON, Canada) according to the manufacturer’s protocol. The diagnosis was confirmed by RT-qPCR, with CT values used to estimate viral load. Library preparation and complete genome sequencing were performed according to the instructions provided for the Illumina CovidSeq Test (Illumina, San Diego, CA, USA). However, they were adapted by replacing the SARS-CoV-2 primer pools with primers provided by the sequencing and subtyping protocol for Influenza A and B viruses [27]. Library quantification was performed using the Qubit dsDNA HS Assay on a Qubit 2.0 device (Invitrogen, Waltham, MA, USA). Quality control of the libraries was verified using a TapeStation 4150 system and a High Sensitivity D1000 ScreenTape kit (Agilent Technologies, Santa Clara, CA, USA). Sequencing was performed using a MiSeq Reagent Kit v2 (2 × 150 cycles) (Illumina, San Diego, CA, USA). The NextSeq PhiX Control kit was used as a normalization sample, and the libraries were sequenced on the MiSeq system (Illumina, San Diego, CA, USA).

Only the brain swab sample of the Brown Booby (*Sula leucogaster*) yielded sufficient material for complete sequencing. The resulting Fastq files represented demultiplexed, raw sequencing data. Quality assessment and trimming of raw reads, including removal of low-quality reads (Phred score < 30), duplicate sequences, adapters, and primers from library construction, were performed using Geneious Prime software 2019.1.3 (https://www.geneious.com, accessed on 1 June 2026). Filtered reads were then mapped to the reference genome A/Thalasseus acuflavidus/EspiritoSanto/1339_N2/2023(H5N1) (NCBI accession numbers: OR269884.1 to OR269891.1) using the Map to Reference tool in Geneious Prime 2019.1.3 (Boston, MA, USA). Finally, the consensus samples were analyzed for the greatest similarity using BlastX (https://blast.ncbi.nlm.nih.gov/Blast.cgi, accessed on 1 June 2026).

### 2.8. Data Analysis

Excel 2016 (Microsoft Office 365) was used for data collection and organization. Descriptive analysis was performed to evaluate nominal variables, expressed as frequencies and percentages for species and AIV positivity.

To estimate the positivity rate of AIV in the collected samples, the minimum sample size was calculated using the formula proposed by Thrusfield et al. (2017) for a theoretically infinite population [28]. The calculation assumed an expected AIV prevalence of 5% [29], and a desired absolute precision of 5%, resulting in a minimum required sample size of 73 individuals. The 95% confidence interval (CI 95%) was estimated using the Wilson score method, assuming a binomial distribution of proportions [30].

### 2.9. Phylogenetic and Phylogeographic Analyses

All available Brazilian AIV HA gene sequences were retrieved from the NCBI GenBank database. Only sequences that met the following criteria were included in the analyses: (i) complete or near-complete HA gene sequences; (ii) samples collected in Brazil between 2022 and 2025; (iii) avian hosts; and (iv) availability of complete metadata, including sampling date and sampling location. Sequences lacking precise collection dates, host information, or geographic origin were excluded from the dataset. For phylogeographic analyses, we only retained only samples with sufficiently detailed locality information that allowed geographic coordinates to be assigned. Geographic coordinates were assigned based on the reported municipality of collection using Google Earth Pro (Google LLC, Mountain View, CA, USA) and subsequently used in continuous phylogeographic reconstructions. Because continuous phylogeographic approaches reconstruct the spatial diffusion of viral lineages over time and space, accurate temporal and geographic information is required to infer dispersal routes and transmission dynamics. This filtering strategy was adopted to maximize the accuracy of temporal and spatial inferences while minimizing uncertainty due to incomplete epidemiological metadata.

The assembled genome obtained in this study was aligned with a dataset containing 31 Brazilian HA gene sequences, including the genome generated in this study, and one Colombian sequence representing the first detection of H5N1 in South America. All sequences retrieved from the GenBank NCBI database are available at Table S1 [31]. The alignment was conducted using MAFFT v. 7.520 [32]. The resulting alignment was inspected, and the ends were manually trimmed using AliView v. 1.28 [33]. As an initial step, a maximum likelihood (ML) tree was reconstructed using IQ-TREE 2 v. 2.2.2.3 [34], with the best nucleotide substitution model inferred using the Bayesian information criterion in ModelFinder [35]. Branch reliability was tested using ultrafast bootstrap approximation (UFBoot) [36] and SH-like approximate likelihood ratio test (SH-aLRT), each with 10,000 replicates. The phylogenetic tree was visualized in RStudio v4.3.3 [37] using the ggtree package [38]. The temporal signal was evaluated using the root-to-tip regression analysis implemented in TempEst v1.5.1 [39].

Given that the preliminary evaluation performed in TempEst v1.5.1 indicated a strong temporal signal in the dataset, we conducted a continuous phylogeographic analysis using a Bayesian Markov chain Monte Carlo (MCMC) framework implemented in BEAST v1.10.4 [40]. Sequence evolution was modeled using the HKY+I nucleotide substitution model, selected as the best-fit by ModelFinder based on the Bayesian Information Criterion (BIC) (Table S2) [35]. Temporal estimates were obtained under an uncorrelated lognormal relaxed molecular clock [41,42,43]. A CTMC reference prior was assigned to the mean clock rate (ucld.mean), allowing the evolutionary rate to be estimated directly from the data. The initial clock rate was set to 4.45 × 10^−3^ substitutions/site/year, based on an independent preliminary root-to-tip regression analysis performed in TreeTime v. 0.9.3 using the ML tree [44]. This value is consistent with previously reported evolutionary rates for Influenza A viruses, particularly for the HA gene [41,45].

To account for temporal changes in viral population size, a Bayesian Skyline coalescent model with five population groups was used as the tree prior. Spatial diffusion dynamics were reconstructed using a relaxed random walk (RRW) diffusion model with a Cauchy distribution and a jitter window of 0.01 [46,47]. MCMC chains were run for 100 million generations, with parameters and trees sampled every 10,000 steps. Convergence and mixing were assessed in Tracer v1.7.2 by ensuring effective sample size (ESS) values greater than 200 for all parameters [48]. Posterior estimates were summarized after removing the first 10% of MCMC samples as burn-in. Maximum clade credibility (MCC) trees were generated using TreeAnnotator v1.10.4 [40].

The maximum clade credibility (MCC) tree and the posterior distribution of phylogeographic trees were processed in R v. 4.3.3 [37]. Spatiotemporal diffusion histories were extracted from the posterior tree distribution using functions implemented in the SERAPHIM package. MCC branch coordinates and dispersal times were further parsed using custom R functions adapted from the SERAPHIM workflow [49]. Maps were generated in R using ggplot2, sf, natural earth, and Brazilian administrative boundaries obtained from IBGE, allowing the visualization of inferred dispersal routes and spatial spread patterns of H5N1 across Brazil [50,51].

## 3. Results

### 3.1. Samples

A total of 346 birds from São Paulo State were analyzed, accounting for 52.3% of all animals included in the study. These birds yielded 692 swab samples, comprising 346 oropharyngeal and 346 cloacal swabs. Regarding the scope of the avifauna approach, 19 orders, 25 families, and 53 avian species were identified (Table 1). Furthermore, the development stage of the animals collected was variable, with adults (75.1%, *n* = 260/346), juveniles (19.9%, *n* = 69/346), and chicks (4.9%, *n* = 17/346). The white-eyed parakeet (*Psittacara leucophthalmus*), peach-fronted conure (*Eupsittula aurea*), and picazuro pigeon (*Patagioenas picazuro*) were the most collected birds, representing 19.0% (66/346), 6.6% (23/346) and 6.0% (21/346) of sampled species, respectively. The samples analyzed from the northwest region of São Paulo were only from avian species (Table 1).

**Figure 1:**
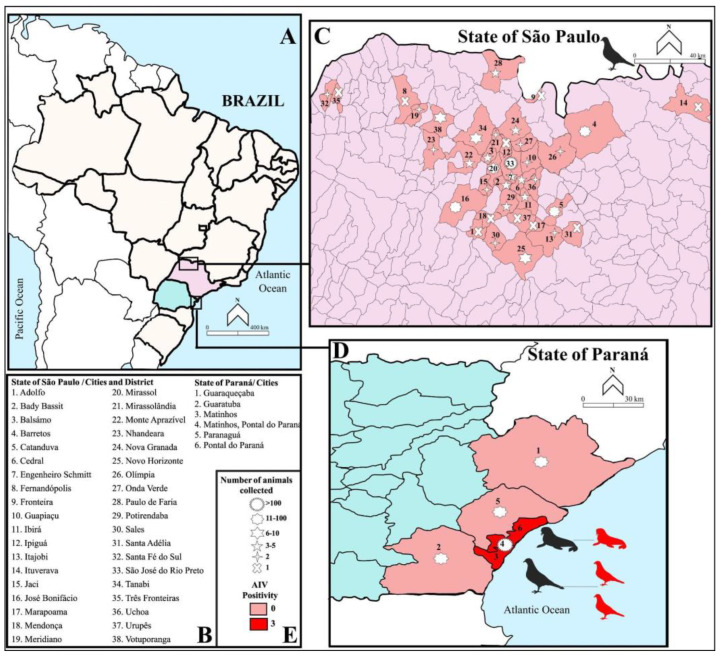
(**A**) The geographical locations of the cities where animals were collected in the northwest region of São Paulo and along the coast of Paraná, Brazil. (**B**) The 38 sampled cities in São Paulo and the 5 in Paraná. (**C**) The sampled cities in São Paulo with their respective sample counts. (**D**) The positive animals and sample collection status for AIV detection between the cities of Matinhos and Pontal do Paraná, Paraná state. (**E**) The number of individuals sampled at each location and the number that tested positive for AIV.

In our analyses, a total of 37 municipalities from northwestern São Paulo and one from Minas Gerais state were sampled (Figure 1A–C). The cities with the highest numbers of samples were São José do Rio Preto (58.9%, n = 204/346), Catanduva (4.6%, *n* = 16/346), and José Bonifácio (3.4%, *n* = 12/346) (Table S3).

Along the Paraná coast, 315 specimens (i.e., birds and mammals) were examined, representing eight orders, 17 families, and 42 identified species. In total, 757 swab samples were evaluated, including avian, marine reptiles, and mammalian species (310 oropharyngeal, 309 anal, 132 encephalic, three nasal, one genital, one pulmonary, and one pancreas). The most collected species were Kelp gull (*Larus dominicanus*), Magellanic penguin (*Spheniscus magellanicus),* and Brown booby (*Sula leucogaster*) (Table 2), representing 21.5% (68/315), 17.1% (54/315), and 16.8% (53/315), respectively. Unlike the samples of live avian specimens sent by the Zoobotanical Garden, the samples from the Paraná coast included live specimens (43.4%, *n* = 137/315), carcasses (53.9%, *n* = 170/315), and animals that were found alive but subsequently died (2.5%, *n* = 8/315).

The animals collected on the coast of Paraná are from six cities. Most of the animals collected were found on the border between Matinhos and Pontal do Paraná (33.0%, *n* = 104/315), followed by Pontal do Paraná (21.2%, *n* = 67/315) and Guaratuba (14.2%, *n* = 45/315). In a few cases, the collection location was not identified (1.5%, n = 5/315) (Figure 1A,B,D; Table S4). The majority of the animals were juveniles (51.7%, *n* = 163/315) or adults (36.8%, *n* = 116/315), with some offspring collected (3.8%, *n* = 12/315), and some individuals could not be identified (7.6%, *n* = 24/315). For sea turtles, only juveniles were evaluated. Altogether, from all of the avian collection regions, 12 species were classified as endangered (MMA Ordinance No. 444/2022) and 20 were migratory.

Finally, regarding the analysis of the endogenous control gene beta-actin, 533 of 661 animals tested across both regions were positive, yielding a positivity rate of 80%.

### 3.2. AIV-Positive Samples

After molecular screening (i.e., RT-qPCR) for AIV in all samples, no positive samples were found among animals from the northwestern region of São Paulo. In contrast, three specimens from the coast of Paraná were AIV-positive, showing a positivity rate of 0.45% (CI 95%: 0.16–1.33) for AIV. The positive samples were all collected between the cities of Matinhos and Pontal do Paraná (Figure 1D,E). The samples belonged to two birds and one mammal. The avian specimens tested positive for all samples received (oropharyngeal, cloacal, and brain swabs) and were found in individuals at the decomposition stage (Table 3). Meanwhile, the mammal was positive in only two of the five samples received—lung and brain swabs from the same individual.

Among the animals that tested positive for AIV by RT-qPCR were a Brown booby (*Sula leucogaster*) in an advanced state of decomposition and a Cabot’s tern (*Thalasseus acuflavidus*), which was initially found alive but which succumbed shortly afterward. Lastly, a mammal, an adult South American sea lion (*Otaria flavescens*), was found in an even more advanced state of decomposition. The positive animals did not exhibit gross or histopathological findings that would have led to their classification as suspected AIV cases during routine surveillance. Therefore, if only necropsy and histopathological findings had been considered during routine surveillance, these animals would likely not have been classified as suspected AIVs cases and consequently would not have been prioritized for further molecular characterization.

In the Brown booby, a sexually mature female, hematoxylin and eosin-stained histological sections of the adrenal glands, spleen, heart and great vessels, stomach, and skin showed no significant histological alterations. The cerebellum/brain exhibited autolysis. The liver, gonads, small intestine, large intestine, skeletal muscle, lungs, thyroid glands, and parathyroid glands exhibited autolysis; however, tissue architecture was preserved. The pancreas exhibited enzymatic necrosis. Moderate-to-marked diffuse congestion was observed in the kidneys. Overall, during the necropsy, the carcass was found to be in a stage of mild decomposition, accompanied by a moderate presence of oily residue, pulmonary edema, and renal congestion.

In a sexually mature male Cabot’s tern, histological sections from the adrenal glands, cerebellum/brain, stomach, gonads, small intestine, large intestine, skin, lungs, and kidneys showed no significant histological alterations. The spleen exhibited moderate hemosiderin accumulation within the splenic parenchyma. The bursa of Fabricius showed a mild multifocal granulocytic infiltrate associated with the occasional presence of bacteria interspersed within the tissue. In the heart, intrasarcoplasmic cysts were observed in some cardiomyocytes, compatible with protozoa *Sarcocystis* spp., without associated inflammation. The liver exhibited moderate diffuse hepatocellular atrophy and mild hemosiderin accumulation within Kupffer cells. In the skeletal muscle, multiple intramyocytic cysts compatible with protozoa *Sarcocystis* spp. were observed, without associated inflammation. The pancreas exhibited marked depletion of zymogen granules within the acinar cells. In general, the lesions were consistent with a possible bacterial infectious process, as well as cachexia syndrome and pancreatic atrophy.

At last, necropsy examination of an approximately 12-year-old male Patagonian sea lion revealed the presence of a gastrolith in the stomach, together with solid anthropogenic debris and fragments of nylon fishing nets. However, due to the animal’s advanced stage of decomposition, many samples were unsuitable for testing. The cause of death in this case was classified as undetermined, but the animal had a good body condition, appearing to have died acutely. Although AIV was detected in the collected samples, additional evidence indicates the possible contribution of other factors to the individual’s death. Nevertheless, the possibility of undetected circulation of AIV remains a cause for concern.

Although pancreatic necrosis was observed in the Brown booby, none of the positive animals exhibited the combination of histopathological lesions typically associated with HPAIV H5N1 infection, such as splenic necrosis, encephalitis, neuronal necrosis, myocarditis, myocardial necrosis, respiratory tract necrosis, or multifocal necrosis in multiple organs [14,53].

### 3.3. Whole-Genome Sequencing and Phylodynamic Analysis

Sequencing was performed on all positive samples; however, successful sequencing was achieved only for samples obtained from the Brown booby (*Sula leucogaster*). A total of 925,720 reads were retrieved, allowing the assembly of eight fragments of the AIV genome: PB2 (2405 nt), PB1 (2342 nt), PA (2265 nt), HA (1843 nt), NP (1611 nt), NA (1458 nt), MP (1045 nt), and NS (896 nt), with genome average coverage ranging from 98.8% to 100% per fragment (Figure S1). All the recovered segments showed high similarity with HPAIV H5N1 2.3.4.4b clade, genotype B3.2. The complete genome obtained in this study is available at the EpiFlu-GISAID database (https://www.gisaid.org/, accessed on 1 June 2026) under the accession number EPI_ISL_1897537.

**Figure 2:**
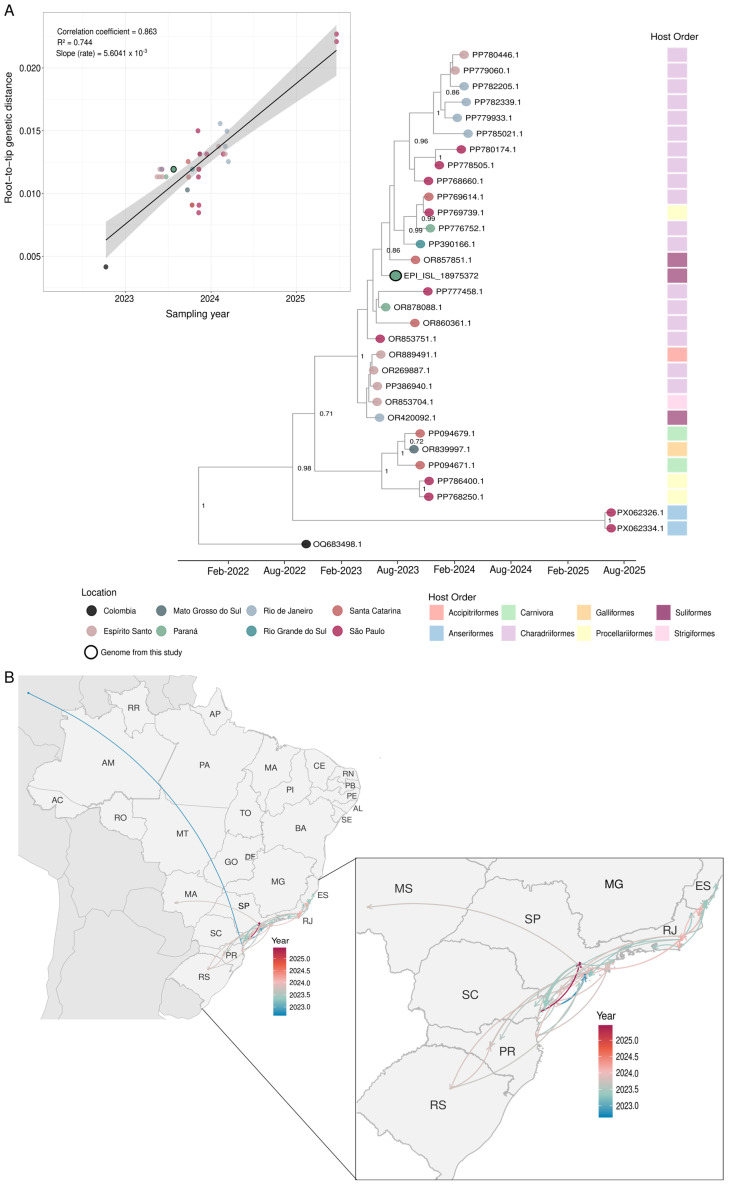
Phylogenetic and phylogeographic reconstruction of H5N1 clade 2.3.4.4b viruses circulating in Brazil. (**A**) Bayesian time-scaled maximum clade credibility (MCC) phylogenetic tree inferred from hemagglutinin (HA) gene sequences, including the H5N1 virus detected in *Sula leucogaster* from the coast of Paraná, southern Brazil (EPI_ISL_18975372; indicated by a black circle), and representative H5N1 clade 2.3.4.4b sequences. The inset shows the root-to-tip regression analysis used to assess the temporal signal of the dataset, depicting the relationship between genetic divergence and sampling time. (**B**) Continuous phylogeographic reconstruction inferred from complete HA gene sequences of H5N1 clade 2.3.4.4b viruses sampled in Brazil between 2022 and 2025, including the genome generated in this study (EPI_ISL_18975372). Colored branches represent the estimated spatiotemporal diffusion of viral lineages through time, illustrating the inferred routes and directionality of H5N1 dissemination across Brazil.

To better understand the spatiotemporal dynamics and spread of HPAIV H5N1, we reconstructed a Bayesian phylogenetic tree based on the HA gene. This analysis included 31 sequences from seven Brazilian states in the Midwest, Southeast, and South regions, as well as one sequence from Colombia, representing the first report of H5N1 in South America (Table S1). All sequences used for phylogenetic analysis were collected between 2022 and 2025 and were detected in distinct wild animals. Root-to-tip regression analysis revealed a strong temporal signal (correlation coefficient = 0.863; R^2^ = 0.744), supporting the suitability of the dataset for time-scaled phylogenetic inference. The estimated evolutionary rate was 5.60 × 10^−3^ substitutions/site/year (Figure 2A).

The Bayesian inference analysis revealed two main clades. The first comprised two sequences collected in 2025, which showed greater genetic divergence relative to the remaining Brazilian sequences, as reflected by their longer branch lengths in the phylogenetic tree and more distant position in the root-to-tip regression analysis. (Figure 2A). Both sequences originated from *Dendrocygna viduata* individuals residing in Ibirapuera Central Park, São Paulo, highlighting the detection of H5N1 in an urban environment and emphasizing the importance of surveillance in urban wildlife. In contrast, the second clade grouped all remaining Brazilian sequences, including the sequence generated in this study. Interestingly, our sequence clustered closely with others from Santa Catarina, which were detected in a Brown booby (*Sula leucogaster*), and a Royal tern (*Thalasseus maximus*) from Rio Grande do Sul, both collected between 2023 and 2024, as well as with sequences from Paraná and São Paulo from a Cabot’s tern (*Thalasseus acuflavidus*) and White-chinned Petrel (*Procellaria aequinoctialis*); these samples were collected in 2023 (Figure 2A). This clustering pattern, characterized by the virus circulating among multiple avian species along the coastal regions of adjacent states, is consistent with viral dissemination mediated by the movement of migratory and coastal bird species. (Figure 2A). Additionally, our phylogenetic analysis further indicates that most Brazilian HA sequences were closely related, exhibiting relatively short genetic distances and a gradual accumulation of divergence through time. Furthermore, based on our dataset, the Bayesian inference indicates that the most recent common ancestor (tMRCA) of HPAIV H5N1, detected in several wild species along the Brazilian coast between 2023 and 2024, was estimated to date back to late 2022 (2022.84; 95% HPD: 2022.36–2023.23), corresponding to November 2022 (95% HPD: May 2022–March 2023).

To further investigate the viral spread, we performed a continuous phylogeographic reconstruction. The analysis suggests that the ancestral lineage was located in southeastern Brazil, followed by dispersal events toward Paraná, Espírito Santo, Rio de Janeiro, Santa Catarina, Rio Grande do Sul, and Mato Grosso do Sul (Figure 2B). We found no evidence supporting a strictly unidirectional dispersal pattern. Instead, viral spread was characterized by multiple movement events among regions, likely associated with host movement along the Brazilian coast (Figure 2B). These findings underscore the importance of continuous monitoring of wild fauna along the Brazilian coastline for the early detection of emerging viral lineages and the tracking of their spatial dissemination.

## 4. Discussion

HPAIV H5N1, belonging to clade 2.3.4.4b, has been detected in numerous countries across Asia, Africa, Europe, and North America since October 2020, leading to the loss of millions of domestic and wild animals [54,55]. By late 2022, the virus had reached South America, with cases reported in Brazil, Colombia, Peru, and Uruguay [5,56,57]. The recent introduction of HPAIV H5N1 in the Americas poses a major threat to wild and endangered species and to poultry production [57]. In addition, the resurgence of HPAIV H5N1 has revealed significant changes in the virus’s ecology and evolution, including an expanded geographic distribution and host range [58]. Notably, there has been an increase in reports of infections in both terrestrial and marine mammals [7]. The capacity of HPAIV H5N1 to infect mammals is a cause for significant public health concern, due to the ongoing potential of AIVs for genetic reassortment, evolution, and widespread dissemination, as varying selective pressures across hosts can facilitate larger outbreaks [59].

In light of this scenario, monitoring HPAIV H5N1 infections is crucial for assessing the extent of viral dissemination to atypical hosts, which is essential for mitigating impacts on biodiversity and public health [60]. Nonetheless, surveillance efforts are often focused on animals exhibiting clinical signs commonly associated with AIV. These signs may include ataxia (inability to walk or stand), tremors, muscle spasms, and respiratory distress [4]. In this study, we observed an AIV detection rate of 0.45%. Three species, two avian and one mammalian, tested positive for the virus, with all specimens collected along the coast of Paraná State from July to October 2023. Among them, only the Cabot’s tern (*Thalasseus acuflavidus*) was found alive at the time of sampling; however, it subsequently died. Interestingly, for this species, no signs indicating HPAIV infection were found during the clinical evaluation.

Previous studies have reported that pathological signals of HPAIV H5N1 infection include pancreatic and splenic necrosis in the avian orders Galliformes and Anseriformes [61]. Additionally, a study involving seals (*Phoca vitulina* and *Halichoerus grypus*) identified inflammation and necrosis in various organ tissues, including the brain, lungs, adrenal glands, liver, and lymph nodes, during post mortem examination [61]. In contrast, none of the animals that tested positive for AIV in this study were initially classified as suspected cases based on clinical or histopathological findings. This highlights the value of including apparently non-suspect animals in surveillance programs to improve the early detection of AIV circulation. Nevertheless, two of the three positive animals were in an advanced state of decomposition, which may have limited the detection and interpretation of pathological lesions. Furthermore, the detection of viral RNA in these samples may also be associated with environmental contamination or exposure through scavenging behavior, including contact with or ingestion of infected carcasses.

Confirming previous reports, our results indicated that the HPAIV H5N1 detected in the Brown booby (*Sula leucogaster*) belongs to the 2.3.4.4b clade (B3.2 genotype). This virus was found along the coast of Paraná, between the cities of Matinhos and Pontal do Paraná. Unfortunately, sequencing data could not be obtained from the other two animals that tested positive for AIV by RT-qPCR, and virus isolation from the Patagonian sea lion was also unsuccessful. Although deceased infected pinnipeds may act as sources of the virus for other species, our study was unable to infer this. This may have been due to the advanced decomposition and low viral concentrations within the samples, which likely influenced the results.

The risk of potential transmission among sea lions is heightened by their gregarious behavior, the large number of affected individuals, and the tendency for deaths to occur in groups [14]. Different routes of infection have been proposed, including both direct transmission between individuals and indirect environmental transmission [57]. Understanding the evolution of HPAIV H5N1 in mammalian hosts, including its capacity to infect and replicate in these species, is essential for assessing the potential for further viral adaptation and the associated risk of zoonotic transmission, which could have implications for future pandemic emergence [62].

The impact of HPAIV H5N1 on marine mammal populations has become increasingly evident through a series of mortality events reported across South America during 2023. In Peru, thousands of Patagonian sea lions died along the coast between January and April 2023 [14]. In January 2023, Chile also reported elevated mortality among sea lions, further heightening concern [63]. In Argentina, sea lion mortality associated with HPAIV H5N1 was reported in August 2023 [4]. In the present study, AIV-positive samples detected by RT-qPCR were obtained from a sea lion that was found dead in Paraná State in October 2023, a state bordering Santa Catarina, where HPAIV H5N1 2.3.4.4b (B3.2) had also been detected in a South American sea lion during the same month [15]. Although viral sequencing was not possible in this study, the temporal and geographic proximity of these detections may be consistent with the regional circulation of HPAIV H5N1 among marine mammals during 2023. The circulation of HPAIV H5N1 in mammalian hosts is of particular importance because it may facilitate viral adaptation and increase zoonotic transmission [64].

A Bayesian phylogenetic analysis was performed, with the root of the tree corresponding to a Colombian sequence, which represents the initial detection of H5N1 clade 2.3.4.4b in South America in 2022 [11,60]. The complete HA sequence identified in this study clustered with sequences from Santa Catarina and Rio Grande do Sul collected between 2023 and 2024, as well as with other sequences from southern and southeastern Brazil. The sequences most closely related to the one obtained in the present study were predominantly derived from avian hosts of the order Charadriiformes, with additional closely related sequences originating from Suliformes. Both avian orders are recognized as well-established reservoirs for this virus [5,15].

The root-to-tip regression analysis showed that most sequences exhibited high similarity, although a progressive divergence was observed over time. The estimated tMRCA of the principal Brazilian clade dates back to approximately November 2022, indicating that the common ancestor of the viruses circulating along the Brazilian coast likely emerged several months before the first detections were reported in the country [65]. Continuous phylogeographic reconstruction inferred the ancestral lineage in southeastern Brazil, followed by dispersal events toward Paraná, Rio de Janeiro, Santa Catarina, Rio Grande do Sul, and Mato Grosso do Sul. Several migratory bird species follow multiple routes that can facilitate the spread of HPAIV H5N1, including migratory pathways through Iceland, Greenland/the Canadian High Arctic, and the Atlantic Ocean [23]. These dispersal patterns are consistent with previous studies suggesting that migratory flyways may contribute to the long-distance spread of HPAIV H5N1 across the Americas [66,67]. Interestingly, the phylogeographic reconstruction did not support a strictly unidirectional dispersal process. Instead, multiple viral movement events were inferred among neighboring coastal states, suggesting repeated exchanges among host populations and highlighting the dynamic nature of H5N1 circulation along the Brazilian coastline. Additionally, the clustering of viruses detected in seabirds and coastal bird species from multiple states further supports the role of marine and coastal ecosystems in maintaining and disseminating HPAIV H5N1 along the Brazilian coast.

Since 2022, several South American countries have reported outbreaks of HPAIV H5N1 [11,68,69,70]. Among the earliest reports, Colombia documented seven outbreaks in a rural area between October and November 2022 [11,68,69]. In November of the same year, Peru registered the death of hundreds of Peruvian Pelicans (*Pelecanus thagus*) and 24 Blue-footed booby (*Sula nebouxii*) [67,68]. Subsequently, in December 2022, Chile confirmed cases in aquatic bird species [71,72]. In February 2023, Uruguay reported detecting the virus in two black-necked swans (*Cygnus melancoryphus*) that were found dead [57]. And then in Brazil, cases were subsequently detected in wild birds in early 2023 [5].

Furthermore, it is essential to emphasize that this study evaluated 12 endangered species, and one was AIV positive (Cabot’s Tern, *Thalasseus acuflavidus*). Negative results are also crucial for maintaining surveillance in health assessments and conservation initiatives. Therefore, these results reinforce the importance of strengthening the One Health approach and of monitoring wild animals, including stranded marine life.

In summary, our findings underscore the importance of molecular characterization in detecting HPAI H5N1, as genomic sequencing enables the reconstruction of viral evolutionary relationships and spatiotemporal dissemination patterns. The literature also reports cases of HPAI H5N1 infection presenting without overt clinical signs but with viral shedding lasting up to 17 days [73]. Therefore, systematic surveillance of both avian and mammalian species using molecular diagnostic techniques is indispensable, even in the absence of clinical or histopathological evidence of AIV infection at the time of sampling. Moreover, mortality events serve as crucial indicators for launching comprehensive investigations, as they may contribute to the persistence of epizootic circulation and promote further transmission events.

## 5. Conclusions

This study confirmed AIV detection via RT-qPCR in three specimens collected along the coast of Paraná, southern Brazil. Samples from a Brown booby (*Sula leucogaster*) confirmed the presence of HPAIV H5N1 clade 2.3.4.4b collected between the municipalities of Matinhos and Pontal do Paraná in 2023. Notably, two birds and one marine mammal that were not classified as suspected AIV cases during monitoring due to the absence of clinical signs and histopathological lesions tested positive via RT-qPCR. These findings highlight the value of including apparently non-suspect animals in surveillance programs, reducing the risk of underestimating viral circulation. Although comprehensive molecular characterization of all recovered animals may not be feasible due to logistical and technical constraints, surveillance protocols could include individuals who do not present as conventional suspects in AIV cases, thereby enhancing the accuracy and scope of epidemiological investigations. The findings of this study also reinforce the need to integrate wildlife health surveillance with conservation strategies under a One Health approach. Furthermore, phylogenetic analysis provided insights into the genetic diversity, clustering, and dispersal of circulating strains, contributing to a better understanding of AIV epidemiology in the region. Future longitudinal surveillance and serological studies are warranted to provide further insights into AIV infection dynamics, transmission pathways, and patterns of viral circulation in wildlife populations.

## Figures and Tables

**Table 1:** Classification and abundance of avian species tested for Avian influenza viruses (AIVs) collected in the northwest region of São Paulo (SP), Brazil, between the years 2022 and 2025. Order: Taxonomic order of the animals. Family: Taxonomic family of the animals. *Scientific name*: Genus and species classification. Number of birds: Total number of animals sampled and number of AIVs. Threatened species: Indicates whether the species is classified as threatened (Yes), not threatened, or not available (NA) in the Brazilian Red List of Threatened Species. Classified as a migratory species: Classification of species as migratory (Yes), non-migratory (No), or not available (NA).

Order	Family	*Scientific Name*	Number of Birds	Threatened Species (Brazilian Red List) [52]	Classified as a Migratory Species [52]
		*-*	1	-	-
Accipitriformes	Accipitridae	*Geranoaetus albicaudatus*	2	No	NA
		*Harpia harpyja*	1	Yes	NA
		*Ictinia plumbea*	1	No	Yes
		*Rupornis magnirostris*	16	No	NA
Anseriformes	Anatidae	*Alopochen aegyptiaca*	1	NA	NA
		*Cairina moschata*	5	No	No
		*Dendrocygna autumnalis*	1	No	No
		*Nomonyx dominicus*	1	No	NA
		*Spatula querquedula*	1	NA	NA
	Anhimidae	*Anhima cornuta*	1	No	No
Caprimulgiformes	Caprimulgidae	*Nyctidromus albicollis*	3	No	NA
Cariamiformes	Cariamidae	*Cariama cristata*	19	No	No
Cathartiformes	Cathartidae	*Coragyps atratus*	19	No	No
Charadriiformes	Charadriidae	*Vanellus chilensis*	2	No	No
Columbiformes	Columbidae	*Columba livia*	8	No	No
		*Columbina*	1	No	No
		*Patagioenas picazuro*	21	No	NA
		*Zenaida auriculata*	2	No	NA
Coraciiformes	Momotidae	*Momotus momota*	1	No	NA
Cuculiformes	Cuculidae	*Guira guira*	1	No	NA
Falconiformes	Falconidae	*Caracara plancus*	10	No	No
		*Falco sparverius*	5	No	NA
Galináceos	Phasianidae	*Pavo cristatus*	1	NA	NA
Nyctibiiformes	Nyctibiidae	*Nyctibius griseus*	2	No	NA
Passeriformes	Icteridae	*Gnorimopsar chopi*	1	No	NA
		*Icterus pyrrhopterus*	1	No	NA
	Thraupidae	*Thraupis sayaca*	1	No	NA
	Tyrannidae	*Colaptes campestris*	1	No	No
		*Pitangus sulphuratus*	4	No	NA
		*Tyrannus melancholicus*	1	No	NA
		*Tyrannus savana*	1	No	NA
Pelecaniformes	Ardeidae	*Ardea alba*	1	No	No
		*Bubulcus-ibis*	3	No	No
		*Butorides striata*	1	No	No
		*Nycticorax nycticorax*	3	No	NA
		*Syrigma sibilatrix*	2	No	No
	Threskiornithidae	*Theristicus caudatus*	8	No	No
Piciformes	Picidae	*Melanerpes candidus*	1	No	NA
	Ramphastidae	*Pteroglossus castanotis*	1	No	No
		*Ramphastos toco*	20	No	No
Psittaciformes	Psittacidae	*Amazona aestiva*	5	Yes	No
		*Amazona amazonica*	2	No	No
		*Ara ararauna*	8	No	No
		*Aratinga auricapillus*	2	No	No
		*Brotogeris chiriri*	19	No	No
		*Eupsittula aurea*	23	No	NA
		*Psittacara leucophthalmus*	66	No	No
Strigiformes	Strigidae	*Athene cunicularia*	16	No	No
		*Megascops choliba*	3	No	NA
		*Pulsatrix perspicillata*	1	No	No
		*Strix virgata*	5	No	NA
	Tytonidae	*Tyto furcata*	18	No	NA
Tinamiformes	Tinamidae	*Rhynchotus rufescens*	1	No	NA
Trochiliformes	Trochilidae	*-*	1	-	-
Total			346		

**Table 2:** Classification and abundance of avian, reptilian, and mammalian species analyzed for avian influenza viruses (AIVs) and sampled along the coast of Paraná (PR), Brazil, between 2022 and 2025. Order: Taxonomic order of the animals. Family: Taxonomic family of the animals. *Scientific name*: Genus and species classification. Number of animals/positives: Total number of animals sampled and number of AIV-positive animals. Threatened species: Indicates whether the species is classified as threatened (Yes), not threatened, or not available (NA) in the Brazilian Red List of Threatened Species. Classified as a migratory species: Classification of species as migratory (Yes), non-migratory (No), or not available (NA). Bold: AIV positive.

Order	Family	*Scientific Name*	Number of Animals/Positive	Threatened Species (Brazilian Red List) [52]	Classified as a Migratory Species [52]
Carnivora	Otariidae	*Arctocephalus australis*	3	NA	No
		*Arctocephalus tropicalis*	2	NA	No
		***Otaria flavescens***	**2/1**	**No**	Yes
Cetacea	Balaenopteridae	*Megaptera novaeangliae*	1	No	Yes
	Delphinidae	*-*	1	-	-
		*Sotalia guianensis*	17	Yes	Yes
		*Stenella frontalis*	1	No	NA
		*Tursiops* sp.	1	-	-
	Hyperoodontidae	*Mesoplodon* sp.	1	-	-
	Odontoceti	*Pontoporia blainvillei*	7	Yes	No
Charadriiformes	Charadriidae	*Charadrius* sp.	1	-	-
		*Pluvialis dominica*	3	No	Yes
		*Pluvialis squatarola*	1	No	Yes
	Laridae	*Chroicocephalus maculipennis*	1	No	No
		*Larus dominicanus*	68	No	NA
		*Rynchops niger*	3	No	Yes
		*Sterna hirundinacea*	1	Yes	Yes
		*Sterna hirundo*	4	No	NA
		***Thalasseus acuflavidus***	**8/1**	**Yes**	**Yes**
		*Thalasseus maximus*	2	Yes	Yes
	Scolopacidae	*Calidris canutus*	5	Yes	Yes
		*Calidris fuscicollis*	1	No	Yes
		*Gallinago paraguaiae*	1	No	No
	Stercorariidae	*Stercorarius chilensis*	1	No	Yes
		*Stercorarius pomarinus*	1	No	NA
		*Stercorarius* sp.	1	-	-
Pelecaniformes	Threskiornithidae	*Phimosus infuscatus*	3	No	No
Procellariiformes	Diomedeidae	*Thalassarche chlororhynchos*	1	Yes	Yes
		*Thalassarche melanophris*	1	Yes	NA
	Procellariidae	*Calonectris* sp.	15	-	-
		*Daption capense*	1	-	Yes
		*Procellaria aequinoctialis*	1	Yes	Yes
		*Pterodroma* sp.	2	-	-
		*Puffinus gravis*	1	No	Yes
		*Puffinus griseus*	1	No	Yes
		*Puffinus puffinus*	14	No	Yes
Sphenisciformes	Spheniscidae	*Spheniscus magellanicus*	54	No	NA
Suliformes	Fregatidae	*Fregata magnificens*	18	No	No
	Phalacrocoracidae	*Phalacrocorax brasilianus*	9	No	NA
	Sulidae	*Sula dactylatra*	1	No	No
		***Sula leucogaster***	**53/1**	**No**	**No**
Testudines	Cheloniidae	*Chelonia mydas*	2	Yes	Yes
Total			315		

**Table 3:** Classification and data of specimens with positive samples for AIV by RT-qPCR of Paraná coast, Brazil. Family: Taxonomic family of the animal. Common name: The name by which the species is commonly known. *Specie*: Taxonomic name of the species. Stage: Refers to the carcass decomposition stage. Month/year: The month and year in which the animal samples were collected. Swab samples: Samples collected from each animal and tested by RT-qPCR were classified as positive (+), negative (−), or not collected (N).

Family	Common Name	*Specie*	Stage	Month/Year	Swab Samples				
					Oral	Cloacal/Anal	Brain	Nasal	Lung
Sulidae	Brown booby	*Sula leucogaster*	3	07/2023	+	+	+	N	N
Otariidae	Patagonian-sea-lion	*Otaria flavescens*	4	10/2023	−	−	+	−	+
Laridae	Cabot’s Tern	*Thalasseus acuflavidus*	2	08/2023	+	+	+	N	N

## Data Availability

The HPAIV H5N1 raw data (fastq files) generated and analyzed in this study is available in the SRA-NCBI database (www.ncbi.nlm.nih.gov/sra, accessed on 26 June 2026), under accession number PRJNA1481291. HPAIV H5N1 consensus sequence used for phylogenetic and phylogeographyc analysis are fully available in the GISAID EpiFlu database (https://gisaid.org/), under the accession number EPI_ISL_1897537. All accession numbers from the sequences used for Bayesian inference are listed in the Supplementary Table S1. Code, alignment and raw data are also available via the Mendeley Data Repository (https://data.mendeley.com/datasets/5gwvp43yx8/1, accessed on 25 June 2026).

## Supplementary Materials

The following supporting information can be downloaded at https://www.mdpi.com/article/10.3390/v18070738/s1, Table S1: Dataset of HA gene sequences from animals detected in Brazil. ID: sequence identifier; Lat.: latitude; Long.: longitude; Host: host species; Order: taxonomic order of the host species; State: state where the sample was collected; Source: source of the sequence. Table S2: Summary of sequence alignment and model selection results. A total of 32 hemagglutinin (HA) gene sequences with 1672 nucleotide sites were analyzed. ModelFinder identified the HKY+F+I nucleotide substitution model as the best fit according to the Bayesian Information Criterion (BIC). Table S3: Cities from which avian species were rescued and sent to the São José do Rio Preto Zoobotanical Garden for veterinary clinical treatment. Table S4: Cities in Paraná State where bird and mammal samples were collected. Figure S1. Read mapping coverage across the eight genomic segments of HPAIV H5N1 2.3.4.4b. Panels A–H correspond, respectively, to segments 1–8 of the viral genome. Mapping was performed against the reference strain Influenza A virus *A/Thalasseus acuflavidus/EspiritoSanto/1339_N2/2023 (H5N1)* (NCBI accession numbers OR269884.1–OR269891.1). FASTA files of the AIV positive sample.

## Abbreviations

The following abbreviations are used in this manuscript:

AIV	Avian influenza viruses.
BSL 3	Biosafety level 3.
Ct	Cycle Threshold.
HA	Hemagglutinin.
HPAIV	Highly Pathogenic Avian influenza viruses.
M1	Matrix protein.
M2	Membrane protein.
NA	Neuraminidase.
NP	Nucleoprotein.
RT-qPCR	Reverse Transcriptase–Quantitative Polymerase Chain Reaction.

## References

[1] Long J.S., Mistry B., Haslam S.M., Barclay W.S. (2019). Host and Viral Determinants of Influenza A Virus Species Specificity. Nat. Rev. Microbiol..

[2] Uyeki T.M., Milton S., Hamid C.A., Webb C.R., Presley S.M., Shetty V., Rollo S.N., Martinez D.L., Rai S., Gonzales E.R. (2024). Highly Pathogenic Avian Influenza A(H5N1) Virus Infection in a Dairy Farm Worker. N. Engl. J. Med..

[3] Verhagen J.H., Fouchier R.A.M., Lewis N. (2021). Highly Pathogenic Avian Influenza Viruses at the Wild-Domestic Bird Interface in Europe: Future Directions for Research and Surveillance. Viruses.

[4] Rimondi A., Vanstreels R.E.T., Olivera V., Donini A., Lauriente M.M., Uhart M.M. (2024). Highly Pathogenic Avian Influenza A(H5N1) Viruses from Multispecies Outbreak, Argentina, August 2023. Emerg. Infect. Dis..

[5] Reischak D., Rivetti A.V., Otaka J.N.P., Domingues C.S., Freitas T.d.L., Cardoso F.G., Montesino L.O., da Silva A.L.S., Malta F., Amgarten D. (2023). First Report and Genetic Characterization of the Highly Pathogenic Avian Influenza A(H5N1) Virus in Cabot’s Tern (*Thalasseus acuflavidus*), Brazil. Vet. Anim. Sci..

[6] Puryear W., Sawatzki K., Hill N., Foss A., Stone J.J., Doughty L., Walk D., Gilbert K., Murray M., Cox E. (2023). Highly Pathogenic Avian Influenza A(H5N1) Virus Outbreak in New England Seals, United States. Emerg. Infect. Dis..

[7] Plaza P.I., Gamarra-Toledo V., Euguí J.R., Lambertucci S.A. (2024). Recent Changes in Patterns of Mammal Infection with Highly Pathogenic Avian Influenza A(H5N1) Virus Worldwide. Emerg. Infect. Dis..

[8] Webster R.G., Bean W.J., Gorman O.T., Chambers T.M., Kawaoka Y. (1992). Evolution and Ecology of Influenza A Viruses. Microbiol. Rev..

[9] Krammer F., Smith G.J.D., Fouchier R.A.M., Peiris M., Kedzierska K., Doherty P.C., Palese P., Shaw M.L., Treanor J., Webster R.G. (2018). Influenza. Nat. Rev. Dis. Prim..

[10] Sautto G.A., Kirchenbaum G.A., Ross T.M. (2018). Towards a Universal Influenza Vaccine: Different Approaches for One Goal. Virol. J..

[11] Ruiz-Saenz J., Martinez-Gutierrez M., Pujol F.H. (2023). Multiple Introductions of Highly Pathogenic Avian Influenza H5N1 Clade 2.3.4.4b into South America. Travel. Med. Infect. Dis..

[12] Xu X., Subbarao K., Cox N.J., Guo Y. (1999). Genetic Characterization of the Pathogenic Influenza A/Goose/Guangdong/1/96 (H5N1) Virus: Similarity of Its Hemagglutinin Gene to Those of H5N1 Viruses from the 1997 Outbreaks in Hong Kong. Virology.

[13] Krammer F., Schultz-Cherry S. (2023). We Need to Keep an Eye on Avian Influenza. Nat. Rev. Immunol..

[14] Gamarra-Toledo V., Plaza P.I., Gutiérrez R., Inga-Diaz G., Saravia-Guevara P., Pereyra-Meza O., Coronado-Flores E., Calderón-Cerrón A., Quiroz-Jiménez G., Martinez P. (2023). Mass Mortality of Sea Lions Caused by Highly Pathogenic Avian Influenza A(H5N1) Virus. Emerg. Infect. Dis..

[15] de Carvalho Araujo A., Cho A.Y., Silva L.M.N., Corrêa T.C., de Souza G.C., Albuquerque A.S., Macagnan E., Kolesnikvoas C.K.M., Meurer R., Vieira J.V. (2024). Mortality in Sea Lions Is Associated with the Introduction of the H5N1 Clade 2.3.4.4b Virus in Brazil October 2023: Whole Genome Sequencing and Phylogenetic Analysis. BMC Vet. Res..

[16] Uhart M.M., Vanstreels R.E.T., Nelson M.I., Olivera V., Campagna J., Zavattieri V., Lemey P., Campagna C., Falabella V., Rimondi A. (2024). Epidemiological Data of an Influenza A/H5N1 Outbreak in Elephant Seals in Argentina Indicates Mammal-to-Mammal Transmission. Nat. Commun..

[17] Burrough E.R., Magstadt D.R., Petersen B., Timmermans S.J., Gauger P.C., Zhang J., Siepker C., Mainenti M., Li G., Thompson A.C. (2024). Highly Pathogenic Avian Influenza A(H5N1) Clade 2.3.4.4b Virus Infection in Domestic Dairy Cattle and Cats, United States, 2024. Emerg. Infect. Dis..

[18] Ly H. (2024). Highly Pathogenic Avian Influenza H5N1 Virus Infections of Dairy Cattle and Livestock Handlers in the United States of America. Virulence.

[19] Lindh E., Lounela H., Ikonen N., Kantala T., Savolainen-Kopra C., Kauppinen A., Österlund P., Kareinen L., Katz A., Nokireki T. (2023). Highly Pathogenic Avian Influenza A(H5N1) Virus Infection on Multiple Fur Farms in the South and Central Ostrobothnia Regions of Finland, July 2023. Eurosurveillance.

[20] Kareinen L., Tammiranta N., Kauppinen A., Zecchin B., Pastori A., Monne I., Terregino C., Giussani E., Kaarto R., Karkamo V. (2024). Highly Pathogenic Avian Influenza A(H5N1) Virus Infections on Fur Farms Connected to Mass Mortalities of Black-Headed Gulls, Finland, July to October 2023. Eurosurveillance.

[21] Bruno A., Alfaro-Núñez A., de Mora D., Armas R., Olmedo M., Garcés J., Garcia-Bereguiain M.A. (2023). First Case of Human Infection with Highly Pathogenic H5 Avian Influenza A Virus in South America: A New Zoonotic Pandemic Threat for 2023?. J. Travel. Med..

[22] Pulit-Penaloza J.A., Brock N., Belser J.A., Sun X., Pappas C., Kieran T.J., Basu Thakur P., Zeng H., Cui D., Frederick J. (2024). Highly Pathogenic Avian Influenza A(H5N1) Virus of Clade 2.3.4.4b Isolated from a Human Case in Chile Causes Fatal Disease and Transmits between Co-Housed Ferrets. Emerg. Microbes Infect..

[23] Caliendo V., Lewis N.S., Pohlmann A., Baillie S.R., Banyard A.C., Beer M., Brown I.H., Fouchier R.A.M., Hansen R.D.E., Lameris T.K. (2022). Transatlantic Spread of Highly Pathogenic Avian Influenza H5N1 by Wild Birds from Europe to North America in 2021. Sci. Rep..

[24] da Fonseca G.F., Broadhurst M.K., Nunes T.Y., Di Domenico M., Cantor M., Domit C. (2024). Spatio-Temporal Variability Among Loggerhead Turtle, Caretta Caretta, Strandings off Southern Brazil. ICES J. Mar. Sci..

[25] Spackman E., Senne D.A., Myers T.J., Bulaga L.L., Garber L.P., Perdue M.L., Lohman K., Daum L.T., Suarez D.L. (2002). Development of a Real-Time Reverse Transcriptase PCR Assay for Type A Influenza Virus and the Avian H5 and H7 Hemagglutinin Subtypes. J. Clin. Microbiol..

[26] Van Borm S., Steensels M., Ferreira H.L., Boschmans M., De Vriese J., Lambrecht B., van den Berg T. (2007). A Universal Avian Endogenous Real-Time Reverse Transcriptase-Polymerase Chain Reaction Control and Its Application to Avian Influenza Diagnosis and Quantification. Avian Dis..

[27] Thomazelli L.M., Araujo J., Oliveira D.B., Sanfilippo L., Ferreira C.S., Brentano L., Pelizari V.H., Nakayama C., Duarte R., Hurtado R. (2010). Newcastle Disease Virus in Penguins from King George Island on the Antarctic Region. Vet. Microbiol..

[28] Thrusfield M., Christley R., Brown H., Diggle P.J., French N., Howe K., Kelly L., O’Connor A., Sargeant J., Wood H. (2018). Veterinary Epidemiology.

[29] Bonilla-Aldana D.K., Calle-Hernández D.M., Ulloque-Badaracco J.R., Alarcón-Braga E.A., Hernández-Bustamante E.A., Cabrera-Guzmán J.C., Quispe-Vasquez S.M., Huayta-Cortez M.A., Benites-Zapata V.A., Rodriguez-Morales A.J. (2024). Highly Pathogenic Avian Influenza A(H5N1) in Animals: A Systematic Review and Meta-Analysis. New Microbes New Infect..

[30] Newcombe R.G. (1998). Two-Sided Confidence Intervals for the Single Proportion: Comparison of Seven Methods. Stat. Med..

[31] National Center for Biotechnology Information GenBank Overview. https://www.ncbi.nlm.nih.gov/genbank/.

[32] Katoh K., Standley D.M. (2013). MAFFT Multiple Sequence Alignment Software Version 7: Improvements in Performance and Usability. Mol. Biol. Evol..

[33] Larsson A. (2014). AliView: A Fast and Lightweight Alignment Viewer and Editor for Large Datasets. Bioinformatics.

[34] Minh B.Q., Schmidt H.A., Chernomor O., Schrempf D., Woodhams M.D., von Haeseler A., Lanfear R. (2020). IQ-TREE 2: New Models and Efficient Methods for Phylogenetic Inference in the Genomic Era. Mol. Biol. Evol..

[35] Kalyaanamoorthy S., Minh B.Q., Wong T.K.F., von Haeseler A., Jermiin L.S. (2017). ModelFinder: Fast Model Selection for Accurate Phylogenetic Estimates. Nat. Methods.

[36] Minh B.Q., Nguyen M.A.T., von Haeseler A. (2013). Ultrafast Approximation for Phylogenetic Bootstrap. Mol. Biol. Evol..

[37] R Core Team(2026) R: A Language and Environment for Statistical Computing. R Foundation for Statistical Computing. https://www.r-project.org/.

[38] Xu S., Li L., Luo X., Chen M., Tang W., Zhan L., Dai Z., Lam T.T., Guan Y., Yu G. (2022). Ggtree: A Serialized Data Object for Visualization of a Phylogenetic Tree and Annotation Data. Imeta.

[39] Rambaut A., Lam T.T., Max Carvalho L., Pybus O.G. (2016). Exploring the Temporal Structure of Heterochronous Sequences Using TempEst (Formerly Path-O-Gen). Virus Evol..

[40] Suchard M.A., Lemey P., Baele G., Ayres D.L., Drummond A.J., Rambaut A. (2018). Bayesian Phylogenetic and Phylodynamic Data Integration Using BEAST 1.10. Virus Evol..

[41] Chen R., Holmes E.C. (2006). Avian Influenza Virus Exhibits Rapid Evolutionary Dynamics. Mol. Biol. Evol..

[42] Drummond A.J., Ho S.Y.W., Phillips M.J., Rambaut A. (2006). Relaxed Phylogenetics and Dating with Confidence. PLoS Biol..

[43] Nelson M.I., Wentworth D.E., Culhane M.R., Vincent A.L., Viboud C., LaPointe M.P., Lin X., Holmes E.C., Detmer S.E. (2014). Introductions and Evolution of Human-Origin Seasonal Influenza a Viruses in Multinational Swine Populations. J. Virol..

[44] Sagulenko P., Puller V., Neher R.A. (2018). TreeTime: Maximum-Likelihood Phylodynamic Analysis. Virus Evol..

[45] Rambaut A., Pybus O.G., Nelson M.I., Viboud C., Taubenberger J.K., Holmes E.C. (2008). The Genomic and Epidemiological Dynamics of Human Influenza A Virus. Nature.

[46] Lemey P., Rambaut A., Welch J.J., Suchard M.A. (2010). Phylogeography Takes a Relaxed Random Walk in Continuous Space and Time. Mol. Biol. Evol..

[47] Dellicour S., Gill M.S., Faria N.R., Rambaut A., Pybus O.G., Suchard M.A., Lemey P. (2021). Relax, Keep Walking—A Practical Guide to Continuous Phylogeographic Inference with BEAST. Mol. Biol. Evol..

[48] Rambaut A., Drummond A.J., Xie D., Baele G., Suchard M.A. (2018). Posterior Summarization in Bayesian Phylogenetics Using Tracer 1.7. Syst. Biol..

[49] Dellicour S., Faria N.R., Rose R., Lemey P., Pybus O.G. (2026). SERAPHIM 2.0: An Extended Toolbox for Studying Phylogenetically Informed Movements. Bioinformatics.

[50] Pereira R., Barbosa R. (2026). Geobr: Download Official Spatial Data Sets of Brazil. https://github.com/ipea/geobr/releases.

[51] Massicotte P., South A. (2026). Rnaturalearth: World Map Data from Natural Earth. https://ropensci.r-universe.dev/rnaturalearth.

[52] ICMBio (Instituto Chico Mendes de Conservação da Biodiversidade) (2025). Sistema de Avaliação do Risco de Extinção da Biodiversidade—SALVE. https://salve.icmbio.gov.br/#/.

[53] Martí-Garcia B., Lean F.Z.X., Núñez A., Majó N. (2025). Natural Infections of Highly Pathogenic Avian Influenza Virus H5N1 in Wild Birds between 2020 and 2023 in the UK: A Retrospective Study with Focus on Microscopic Lesions, Viral Distribution and Neurotropism. Vet. Res..

[54] Günther A., Krone O., Svansson V., Pohlmann A., King J., Hallgrimsson G.T., Skarphéðinsson K.H., Sigurðardóttir H., Jónsson S.R., Beer M. (2022). Iceland as Stepping Stone for Spread of Highly Pathogenic Avian Influenza Virus between Europe and North America. Emerg. Infect. Dis..

[55] Sanogo I.N., Djegui F., Akpo Y., Gnanvi C., Dupré G., Rubrum A., Jeevan T., McKenzie P., Webby R.J., Ducatez M.F. (2022). Highly Pathogenic Avian Influenza A(H5N1) Clade 2.3.4.4b Virus in Poultry, Benin, 2021. Emerg. Infect. Dis..

[56] Leguia M., Garcia-Glaessner A., Muñoz-Saavedra B., Juarez D., Barrera P., Calvo-Mac C., Jara J., Silva W., Ploog K., Amaro L. (2023). Highly Pathogenic Avian Influenza A (H5N1) in Marine Mammals and Seabirds in Peru. Nat. Commun..

[57] Marandino A., Tomás G., Panzera Y., Leizagoyen C., Pérez R., Bassetti L., Negro R., Rodríguez S., Pérez R. (2023). Spreading of the High-Pathogenicity Avian Influenza (H5N1) Virus of Clade 2.3.4.4b into Uruguay. Viruses.

[58] Xie R., Edwards K.M., Wille M., Wei X., Wong S.-S., Zanin M., El-Shesheny R., Ducatez M., Poon L.L.M., Kayali G. (2023). The Episodic Resurgence of Highly Pathogenic Avian Influenza H5 Virus. Nature.

[59] Webby R.J., Uyeki T.M. (2024). An Update on Highly Pathogenic Avian Influenza A(H5N1) Virus, Clade 2.3.4.4b. J. Infect. Dis..

[60] Plaza P.I., Gamarra-Toledo V., Rodríguez Euguí J., Rosciano N., Lambertucci S.A. (2024). Pacific and Atlantic Sea Lion Mortality Caused by Highly Pathogenic Avian Influenza A(H5N1) in South America. Travel. Med. Infect. Dis..

[61] Lean F.Z.X., Vitores A.G., Reid S.M., Banyard A.C., Brown I.H., Núñez A., Hansen R.D.E. (2022). Gross Pathology of High Pathogenicity Avian Influenza Virus H5N1 2021-2022 Epizootic in Naturally Infected Birds in the United Kingdom. One Health.

[62] Lair S., Quesnel L., Signore A.V., Delnatte P., Embury-Hyatt C., Nadeau M.-S., Lung O., Ferrell S.T., Michaud R., Berhane Y. (2024). Outbreak of Highly Pathogenic Avian Influenza A(H5N1) Virus in Seals, St. Lawrence Estuary, Quebec, Canada. Emerg. Infect. Dis..

[63] Ulloa M., Fernández A., Ariyama N., Colom-Rivero A., Rivera C., Nuñez P., Sanhueza P., Johow M., Araya H., Torres J.C. (2023). Mass Mortality Event in South American Sea Lions (*Otaria flavescens*) Correlated to Highly Pathogenic Avian Influenza (HPAI) H5N1 Outbreak in Chile. Vet. Q..

[64] Rolfes M.A., Kniss K., Kirby M.K., Garg S., Reinhart K., Davis C.T., Murray E.L., Wadford D.A., Harriman K., Zhu S. (2025). Human Infections with Highly Pathogenic Avian Influenza A(H5N1) Viruses in the United States from March 2024 to May 2025. Nat. Med..

[65] de Araújo A.C., Silva L.M.N., Cho A.Y., Repenning M., Amgarten D., de Moraes A.P., Malta F., Miller M., Dorlass E.G., Palameta S. (2024). Incursion of Highly Pathogenic Avian Influenza A(H5N1) Clade 2.3.4.4b Virus, Brazil, 2023. Emerg. Infect. Dis..

[66] Newcombe P.B., Nilsson C., Lin T.-Y., Winner K., Bernstein G., Maji S., Sheldon D., Farnsworth A., Horton K.G. (2019). Migratory Flight on the Pacific Flyway: Strategies and Tendencies of Wind Drift Compensation. Biol. Lett..

[67] Siembieda J.L., Johnson C.K., Cardona C., Anchell N., Dao N., Reisen W., Boyce W. (2010). Influenza A Viruses in Wild Birds of the Pacific Flyway, 2005–2008. Vector Borne Zoonotic Dis..

[68] Food and Agriculture Organization (FAO) Emergencia y Respuesta a la Influenza Aviar de Alta Patogenicidad H5N1 en América Central, América del Sur y el Caribe. https://www.fao.org/family-farming/detail/es/c/1736289/.

[69] Pan American Health Organization (2022). Epidemiological Alert—Outbreaks of Avian Influenza and Public Health Implications in the Region of the Americas.

[70] Pan American Health Organization (2025). Epidemiological Update: Avian Influenza A(H5N1) in the Americas Region—24 November 2025.

[71] Godoy M., Oca M.M.d., Caro D., Pontigo J.P., Kibenge M., Kibenge F. (2023). Evolution and Current Status of Influenza A Virus in Chile: A Review. Pathogens.

[72] Ariyama N., Pardo-Roa C., Muñoz G., Aguayo C., Ávila C., Mathieu C., Almonacid L.I., Medina R.A., Brito B., Johow M. (2023). Highly Pathogenic Avian Influenza A(H5N1) Clade 2.3.4.4b Virus in Wild Birds, Chile. Emerg. Infect. Dis..

[73] Hulse-Post D.J., Sturm-Ramirez K.M., Humberd J., Seiler P., Govorkova E.A., Krauss S., Scholtissek C., Puthavathana P., Buranathai C., Nguyen T.D. (2005). Role of Domestic Ducks in the Propagation and Biological Evolution of Highly Pathogenic H5N1 Influenza Viruses in Asia. Proc. Natl. Acad. Sci. USA.

