# Supplementary material for: Genomic Surveillance Uncovers the Silent Spread of Avian Influenza Virus (H5N1 2.3.4.4b) Among Wild Birds and Mammals Along Brazil’s Southern Coast
Source: Viruses. 2026 Jul 3;18(7):738. doi: 10.3390/v18070738 (PMC13431486; doi:10.3390/v18070738)

Genomic Surveillance Uncovers the Silent Spread of Avian Influenza Virus (H5N1 2.3.4.4b) Among Wild Birds and Mammals Along Brazil's Coast

Figure S1. Read mapping coverage across the eight genomic segments of HPAIV H5N1 2.3.4.4b. Panels **A–H** correspond, respectively, to segments 1–8 of the viral genome. Mapping was performed against the reference strain Influenza A virus *A/Thalasseus acuflavidus/EspiritoSanto/1339_N2/2023 (H5N1)* (NCBI accession numbers OR269884.1–OR269891.1).

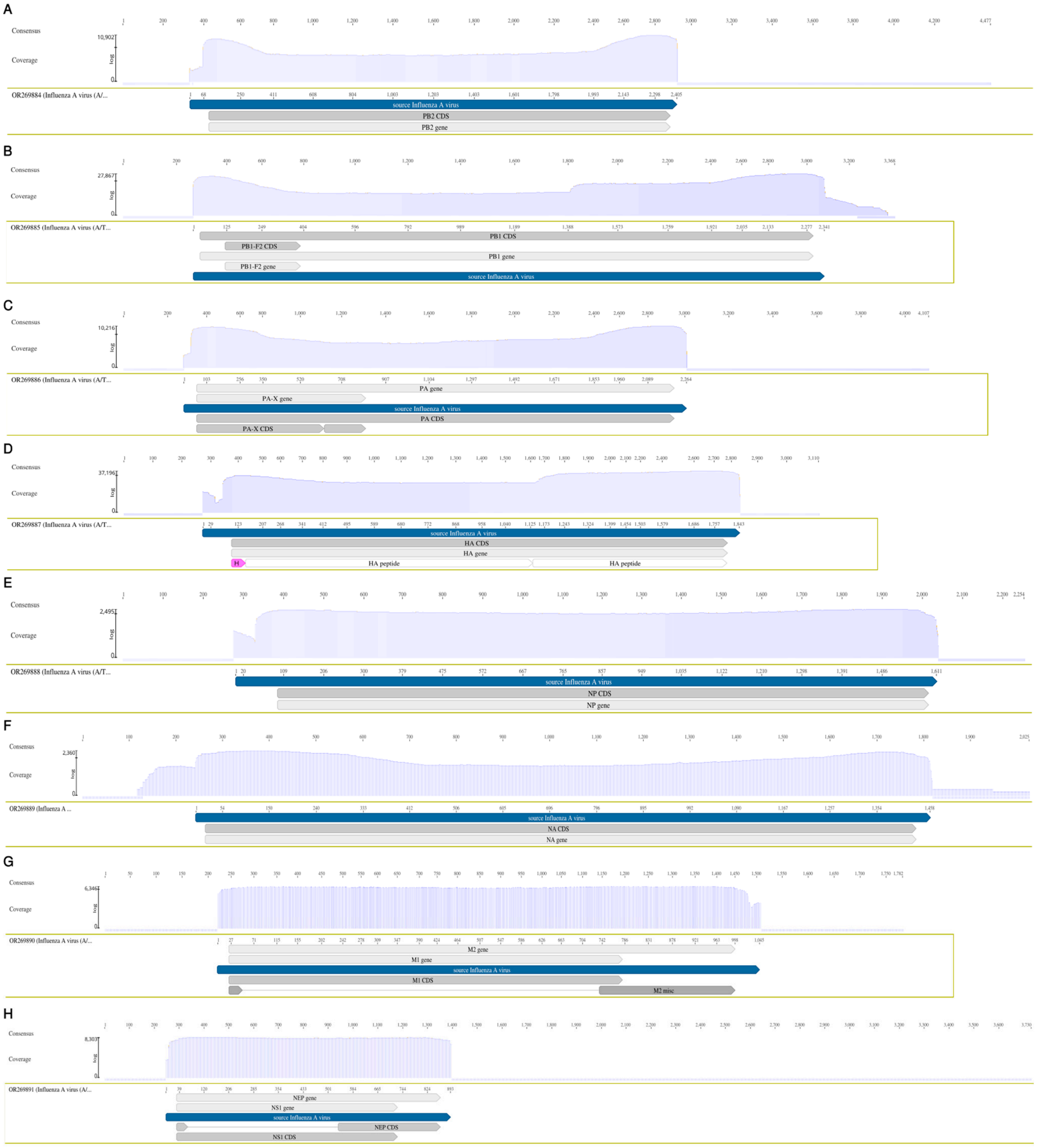

Supplement: Supplementary file 1 [file viruses-18-00738-s001.zip › Suplementary Figure S1.pdf]
