# Supplementary material for: Genomic Surveillance Uncovers the Silent Spread of Avian Influenza Virus (H5N1 2.3.4.4b) Among Wild Birds and Mammals Along Brazil’s Southern Coast
Source: Viruses. 2026 Jul 3;18(7):738. doi: 10.3390/v18070738 (PMC13431486; doi:10.3390/v18070738)
Supplement: Supplementary file 1 [file viruses-18-00738-s001.zip › FASTA files of the AIV positive sample.zip!/Arquivos fasta da amostra positiva/EPI_ISL_18975372 (1).pdf]

GISAID

Isolate detail

Isolate name:	A/Sula_leucogaster/Brazil-PR/A0001-IBTEC/2023
Isolate ID:	EPI_ISL_18975372
Type:	A / H5N1
Passage details/history:	Original
Lineage:	
Clade:	2.3.4.4b
Genotype:	B3.2
Pathogenicity:	HPAI

Sample information

Collection date:	2023-07-25
Location:	Brazil / Estado do Parana / Matinhos
Host:	Wild bird
Additional location information:	
Additional host information:	
Sampling strategy:	
Sequencing strategy:	
Sequencing technology:	
Assembly method:	
Coverage:	
Health status:	Dead
Domestic status:	Wild
Specimen source:	Organ
Vaccinated:	
Strain or commercial product name used for vaccination:	

Institute information

Originating lab:	Laboratorio de Ecologia e Conservação
Address:	Laboratorio de Ecologia e Conservação Av. Beira-mar Pontal do Sul - Pontal do Paraná-PR Brazil
Sample ID given by the sample provider:	
Submitting lab:	São Paulo State University - Biotechnology Institute
Address:	São Paulo State University - Biotechnology Institute Tecomarias Avenue, s/n 18607440 Botucatu Brazil
Sample ID given by the submitting laboratory:	AFLU1
Authors:	Celis, Eliana Leonor Hurtado; Possebon, Fabio Sossai; Garcia, Yasmin Luísa Neves Lemes; Calmon, Marilia Freitas; da Costa, Vivaldo Gomes; de Lima, Fabio Henrique; Domit, Camila; Spilki, Fernando Rosado; Rahal, Paula; Junior, João Pessoa Araújo

In vivo antiviral resistance - Unspecified

Adamantanes:	Unknown
Oseltamivir:	Unknown
Zanamivir:	Unknown
Peramivir:	Unknown

Other: Unknown

In vivo antiviral resistance - Genotype: Unknown

Genotype: B3.2

In vivo antiviral resistance - Phenotype: Unknown

Antigenic characterisation:

Note:

Sequence

segment	identifier	length	accession #
PB2	A/Sula_leucogaster/Brazil-PR/A0001-IBTEC/2023_PB2	2405	
PB1	A/Sula_leucogaster/Brazil-PR/A0001-IBTEC/2023_PB1	2342	
PA	A/Sula_leucogaster/Brazil-PR/A0001-IBTEC/2023_PA	2265	
HA	A/Sula_leucogaster/Brazil-PR/A0001-IBTEC/2023_HA	1843	
NP	A/Sula_leucogaster/Brazil-PR/A0001-IBTEC/2023_NP	1611	
NA	A/Sula_leucogaster/Brazil-PR/A0001-IBTEC/2023_NA	1458	
MP	A/Sula_leucogaster/Brazil-PR/A0001-IBTEC/2023_MP	1045	
NS	A/Sula_leucogaster/Brazil-PR/A0001-IBTEC/2023_NS	896	

Submitter information

Submitter: Hurtado, Eliana

Submission Date: 2024-03-12

Last modifier: Hurtado, Eliana

Last modified: 2024-08-10

Address: São Paulo State University - Biotechnology Institute Tecomarias Avenue, s/n 18607440 Botucatu Brazil
